# Epidemiological Characteristics of Custodial Deaths: An Autopsy Study at a Tertiary Care Institute in Rishikesh

**DOI:** 10.7759/cureus.91737

**Published:** 2025-09-06

**Authors:** Amit Jangid, Arushi Verma, R Sivasankary, Raviprakash Meshram, Shailesh V Parate, Anubhuti Tyagi

**Affiliations:** 1 Forensic Medicine and Toxicology, All India Institute of Medical Sciences, Rishikesh, Rishikesh, IND

**Keywords:** custodial death, jail inmate, negligent death, preventable death, prison healthcare, prison mortality

## Abstract

Background

Custodial death remains a sensitive and critical human rights issue worldwide. Identifying the causes and contributing factors is essential for developing effective interventions and ensuring accountability and dignity for individuals deprived of liberty.

Methods

This retrospective study analyzed records of custody-related deaths examined by the Department of Forensic Medicine, All India Institute of Medical Sciences (AIIMS), Rishikesh. A total of 34 autopsies were conducted between January 2020 and May 2025. The data were organized, coded, and charted, then analyzed using IBM SPSS Statistics for Windows, Version 23.0 (Released 2015; IBM Corp., Armonk, NY, USA) and compared with findings from other national and international studies. Ethical approval was obtained from the Institutional Ethics Committee.

Results

From 2020 to May 2025, 34 custodial deaths were documented, with the highest number (10 cases) reported in 2023. Most of the deceased were men (91%) and over 40 years old (76%), suggesting increased vulnerability with advancing age. No deaths occurred inside jail; the majority took place at AIIMS, Rishikesh (62%), or during transport (26%), indicating delays or inadequacies in medical care. Although 91% of the deaths were classified as natural, many were linked to preventable conditions such as coronary artery disease and chronic liver disease. Notably, the legal status of nearly half of the deceased was undocumented, revealing major deficiencies in custodial record-keeping and accountability.

Conclusions

The increasing number of custodial deaths, predominantly among older male inmates, underscores inadequate healthcare and poor documentation in custody. Most deaths occurred outside jail settings, pointing to delays in medical intervention. The absence of legal status records for nearly half of the deceased highlights systemic shortcomings that demand urgent reform.

## Introduction

Custodial deaths represent a serious human rights concern worldwide. Public perception often associates these deaths with police brutality and torture; however, they may also result from neglect and inhumane treatment within jail custody, a dimension that receives comparatively less attention. In India, custodial deaths are not uncommon [1]. Such cases frequently attract significant public attention and generate controversy, particularly regarding their causes, with media coverage sometimes fueling speculation [2]. Each custodial death should prompt a comprehensive, fair, and unbiased investigation.

The causes of custodial deaths vary widely across countries and regions, shaped by differences in legal systems, institutional practices, and sociopolitical contexts. Autopsy-based analyses from North Indian tertiary care centers remain scarce, especially concerning delayed medical care and comorbidities in custodial settings.

This study aims to investigate the causes of custodial deaths, examine contributing factors, and identify areas where preventive measures and interventions are urgently needed. Understanding these dimensions is essential for safeguarding human rights and ensuring accountability within custodial institutions. The findings are intended to inform systemic reforms and reinforce constitutional protections under Article 21, the right to life for individuals deprived of liberty.

## Materials and methods

This retrospective study was conducted in Rishikesh at a prominent tertiary care institute in North India, which provides healthcare services to the surrounding population. The study period extended from January 2020 to May 2025, during which autopsies were performed on 34 deceased prisoners. These individuals were inmates from the local jail and included those who died while in police or judicial custody, as well as those transferred from jail to the hospital for medical treatment and who subsequently died. In compliance with Indian legal requirements, all deceased individuals underwent medicolegal autopsy.

Epidemiological information was obtained primarily from the inquest papers accompanying the deceased inmates brought for medicolegal autopsy. Additional details were gathered from police and jail authorities, as well as from the relatives of the deceased. The study excluded deaths occurring in the community, hospital patients not under prison or police authority, and incomplete or ambiguous cases where custodial status could not be confirmed.

The collected data were compiled in Excel (Microsoft Corporation, Redmond, WA, USA) and analyzed statistically using IBM SPSS Statistics for Windows, Version 23.0 (Released 2015; IBM Corp., Armonk, NY, USA), and compared with findings from other national and international studies to identify patterns and contributing factors associated with custodial deaths. Ethical clearance was obtained from the Institutional Ethics Committee of All India Institute of Medical Sciences (AIIMS) Rishikesh (approval number AIIMS/IEC/25/383). Since the study involved deceased individuals and relied on official medicolegal records, the requirement for informed consent was waived, in accordance with national ethical guidelines on research involving the dead [3].

## Results

**Figure 1:**
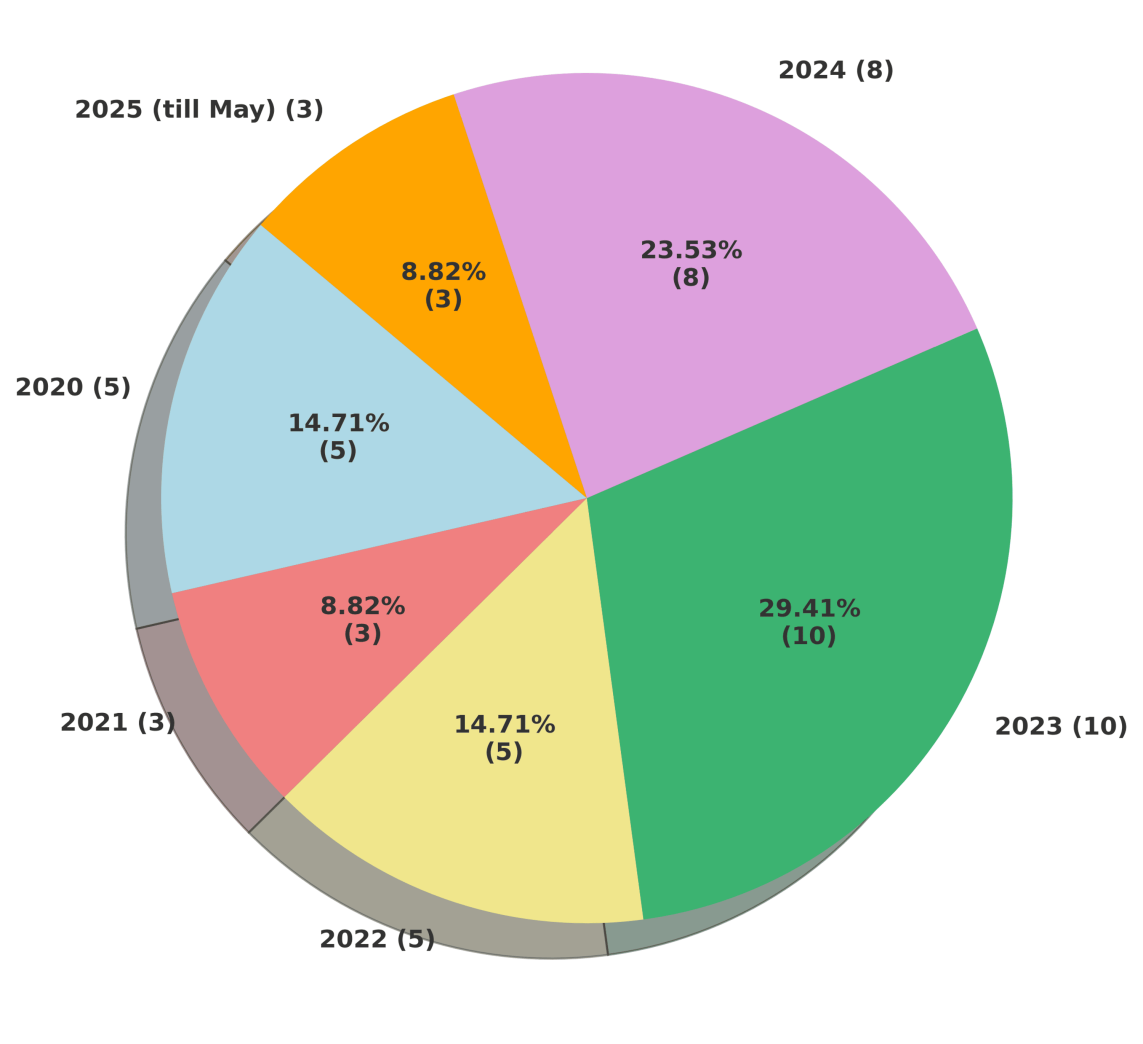
Year-wise distribution of custodial deaths (n = 34)

A total of 34 custodial deaths were analyzed between January 2020 and May 2025, showing a gradual increase in reported cases over time. In both 2020 and 2022, five deaths were reported (15% each), while 2021 recorded a slight decline with three cases (9%). The highest number of deaths occurred in 2023, accounting for 29% (10 cases), followed by 2024 with eight cases (24%). In the first five months of 2025, three deaths (9%) were reported. This upward trend suggests either improved reporting, worsening custodial conditions, or a combination of both. A graphical representation of this trend is shown in Figure 1.

**Figure 2:**
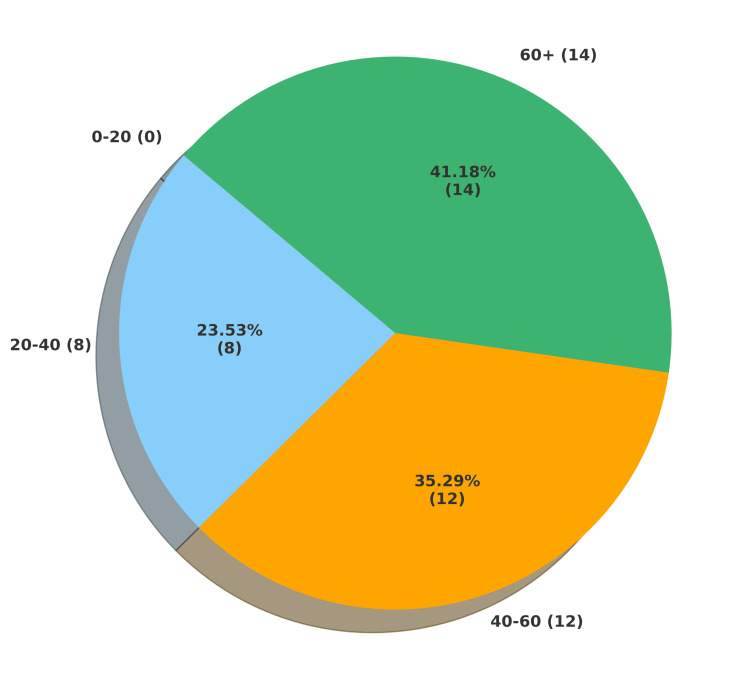
Age-wise distribution of custodial deaths during the study period This figure shows how the frequency of custodial deaths varied across different age groups during the study period.

Most deaths occurred among middle-aged and elderly inmates. No deaths were recorded among individuals aged 0-20 years. Inmates aged 40-60 years accounted for 35.29% of cases, while those aged 60 years and above represented the highest proportion, constituting 41.17%. Nearly three-quarters of all deaths occurred in individuals over 40 years old, underscoring increased vulnerability with age. A graphical representation of the age distribution is provided in Figure 2.

**Figure 3:**
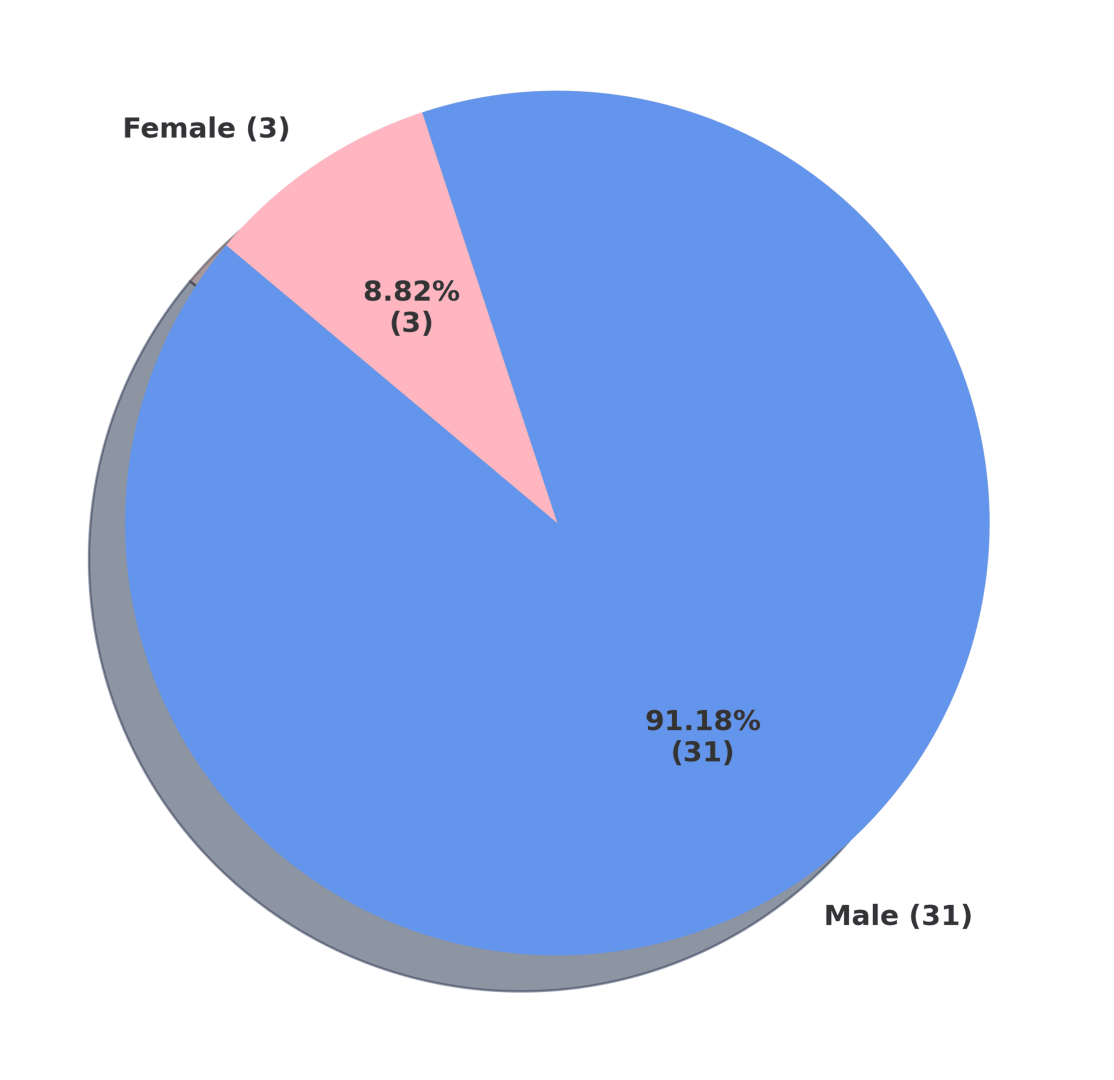
Sex-wise distribution of custodial deaths (n = 34)

The vast majority of the deceased were male, accounting for 31 of the 34 cases (91.17%). Only three female deaths (8.82%) were recorded, reflecting a marked gender imbalance. The detailed sex-wise distribution is presented in Figure 3.

**Figure 4:**
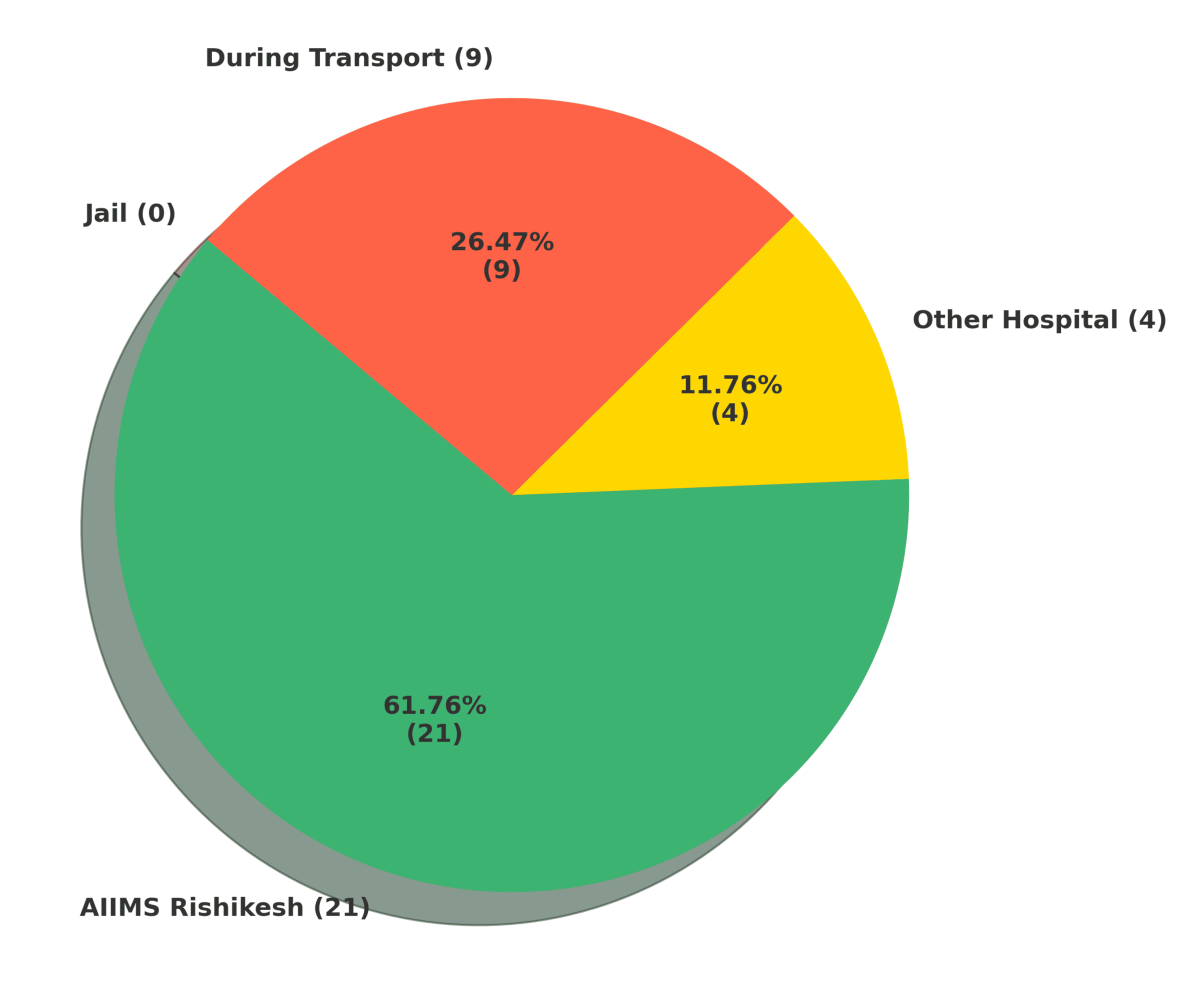
Place-wise distribution of custodial deaths Most custodial deaths occurred in hospitals (primarily AIIMS, Rishikesh), followed by deaths during transport. AIIMS, All India Institute of Medical Sciences

Interestingly, in the present study, none of the deaths occurred directly within jail premises. Most deaths were recorded at AIIMS, Rishikesh, accounting for 21 cases (61.76%). Four individuals (11.76%) died in other hospitals, while nine (26.47%) died during transportation. These findings highlight systemic issues related to delayed or inadequate access to timely medical care for custodial inmates. The distribution of the place of death is illustrated in Figure 4.

**Figure 5:**
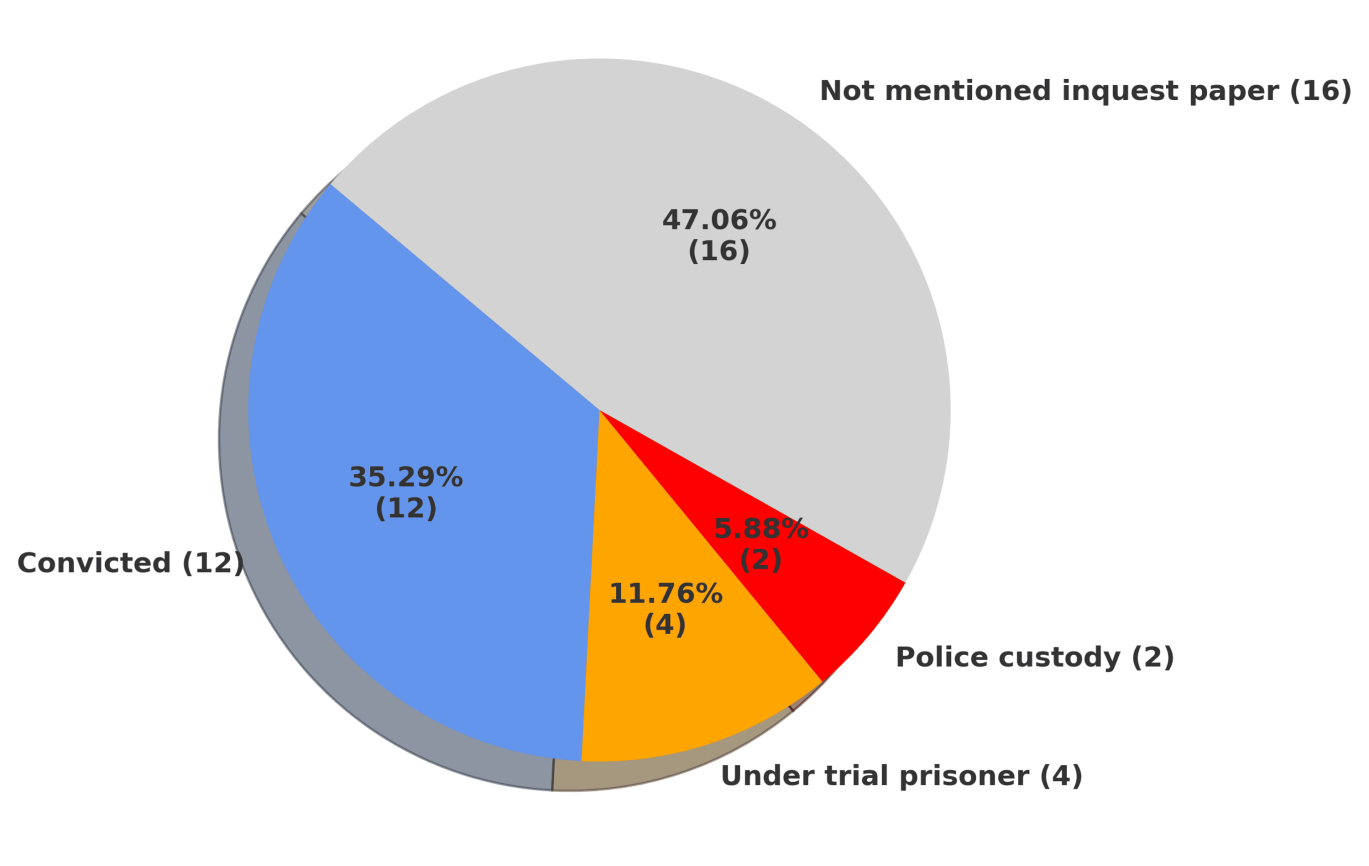
Distribution of custodial deaths (n = 34) by legal status Categories include convicted prisoners, undertrial prisoners, police custody, and unknown legal status.

Among the deceased, 12 individuals (35.29%) were convicted prisoners, while four (11.76%) were undertrial prisoners. Two deaths (5.88%) occurred in police custody. Notably, for 16 individuals (47.05%), the legal status was not mentioned in the inquest papers, reflecting a serious gap in documentation and procedural transparency. The classification of custodial deaths based on legal status is illustrated in Figure 5.

**Figure 6:**
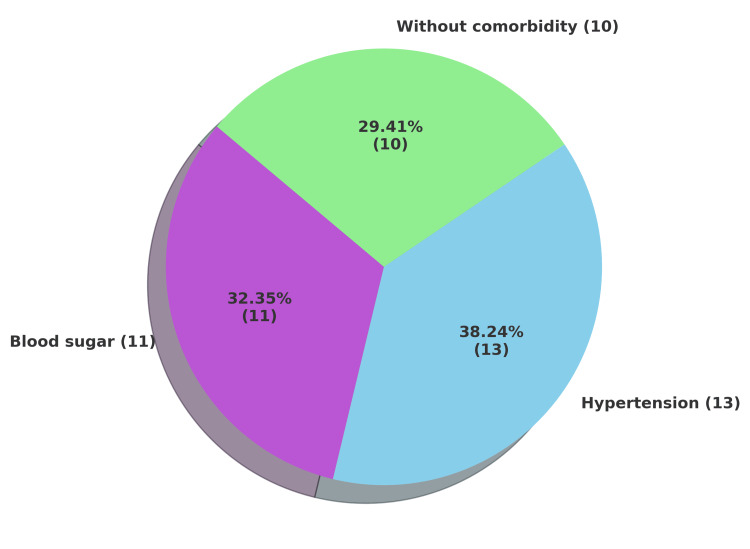
Prevalence of preexisting diseases (n = 34)

With respect to preexisting diseases, 13 individuals (38.23%) had hypertension, and 11 (32.35%) had diabetes. Only 10 cases (29.41%) had no known comorbidities. The distribution of comorbidities is presented in Figure 6.

In the present study, most deaths, 31 out of 34 cases (91.17%), were classified as natural. Three deaths (8.82%) were categorized as unnatural, with causes attributed to blunt trauma during confrontation or injuries sustained prior to arrest.

Among the natural deaths, the leading medical cause was coronary artery disease (CAD), accounting for nine cases (26.4%), followed by chronic liver disease in four cases (11.76%) and septicemia secondary to various conditions in eight cases. COVID-19 contributed to two deaths (5.88%), while pneumonia was identified in one case (2.94%). Other isolated causes included brain injury, hypertrophic cardiomyopathy, lung carcinoma, tuberculosis, spinal injury, intestinal and esophageal perforation, chronic lung disease, and meningoencephalitis.

The broad classification of deaths into natural and unnatural categories is summarized in Table 1, while the detailed distribution of specific medical causes is presented in Table 2.

**Table 1:** Distribution of cases according to cause of death

Cause of death	Number of cases (n)	Percentage (%)
Natural	31	91.17
Unnatural	3	8.82

**Table 2:** Classification and specific medical causes of custodial deaths (n = 34) CAD, coronary artery disease

Causes of death	Number of cases (n)	Percentage (%)
Brain injury	2	5.88
CAD	9	26.4
Hypertrophic cardiomyopathy	1	2.94
COVID-19	2	5.88
Pneumonia	1	2.94
Lung carcinoma	1	2.94
Chronic liver disease	4	11.76
Chronic liver disease (septicemia)	3	8.82
Esophageal mass (septicemia)	1	2.94
Chronic lung disease (septicemia)	3	8.82
Intestinal perforation (septicemia)	3	8.82
Spinal injury	1	2.94
Esophageal perforation (septicemia)	1	2.94
Tuberculosis	1	2.94
Meningoencephalitis	1	2.94

## Discussion

Custodial death refers to the death of an individual while in official custody, such as in a police lock-up, jail, or prison [4]. It also includes deaths that occur during attempts by law enforcement or prison authorities to detain a suspect, or when a person escapes or attempts to escape from custody. Custody denotes care or guardianship [5]. Human rights activists have consistently raised concerns about custodial deaths, emphasizing that every individual is entitled to the fundamental right to life, as guaranteed under Article 21 of the Constitution of India [6]. This right extends beyond mere survival to encompass the right to live with dignity, even while in custody [7,8]. Once the state deprives a person of liberty, it assumes full responsibility for safeguarding their human rights. This includes the duty to respect and ensure the right to life of every individual in custody and obligates law enforcement agencies to provide food, shelter, and medical care [6].

Recent statistics underscore the gravity of the issue. According to data released by the Ministry of Home Affairs and the National Human Rights Commission (NHRC) for the financial year 2021-2022, there were 2,150 deaths in judicial custody and 155 deaths in police custody across various states and union territories of India. Uttarakhand alone reported 27 custodial deaths. These figures, based on National Crime Records Bureau (NCRB) reports, highlight the urgent need for systemic reforms and stronger safeguards to protect the rights of individuals in custody [6,9].

A significant limitation in custodial death investigations is the conflict of interest when another police officer from the same department conducts the inquiry. Even if the officer is not directly involved, there may be risks of bias or attempts to protect colleagues. To ensure impartiality, Section 196(3) of the Bharatiya Nagarik Suraksha Sanhita, 2023, makes it mandatory for a judicial magistrate to conduct an inquest in all cases of custodial death, disappearance, or rape in custody. This provision helps maintain neutrality and public confidence in the investigation process [10].

Autopsies of custodial deaths in India are typically performed by a board of doctors, including a forensic expert. Forensic medicine experts at teaching hospitals of government medical colleges are recommended to conduct these autopsies. Under no circumstances should an autopsy be performed without natural light. If carried out in district or government hospitals, efforts should be made to include a forensic medicine expert. National and state guidelines also encourage videography of the entire procedure to enhance transparency and trust [11].

India’s official prison statistics classify prisoner deaths as either natural (resulting from illness or aging) or unnatural (including suicide, homicide, or accidents) [12,13]. However, the NCRB’s distinction between natural and unnatural causes is often ambiguous. For example, if a prisoner dies due to inadequate medical care or delayed intervention, it is unclear how such a death should be categorized [14]. Any custodial death is a serious concern, particularly when deemed unnatural. In such cases, a thorough investigation is essential, not only to determine the cause and circumstances of death but also to address the concerns and suspicions of the deceased’s relatives regarding events within the police or prison facility [15].

In this study, we examined custodial death cases that occurred between January 2020 and May 2025. Five custodial deaths were reported in 2020. Numbers fluctuated in subsequent years, peaking in 2023 with 10 deaths (29.41%), before declining to eight in 2024 and three in the first five months of 2025. Age-wise distribution revealed that most deaths occurred among individuals over 40 years, with 35.29% in the 40-60-year group and 41.17% in those aged 60 years and above. The most affected subgroup was 51-60 years, a finding consistent with other studies, highlighting the increased vulnerability associated with aging [15,16]. This trend may be explained by the predominance of natural deaths in this cohort, often associated with preexisting diseases and comorbidities that worsen in later decades and remain untreated for years.

The vast majority of the deceased were male, accounting for 31 of the 34 cases (91.17%). Only three female deaths (8.82%) were recorded, reflecting a strong gender imbalance. Like other authors, we observed male predominance in our study. This is often attributed to men being more frequently involved in criminal activities than women, which in itself explains the higher proportion of male custodial deaths [15-19].

Interestingly, none of the deaths in the present study occurred directly within jail premises. Most deaths were recorded at AIIMS, Rishikesh, accounting for 21 cases (61.76%). Four individuals (11.76%) died in other hospitals, and nine (26.47%) died during transportation, likely while being moved for medical treatment or legal proceedings. These findings point toward systemic issues of delayed or inadequate access to medical care. The location of death often reflects institutional practices and healthcare access rather than the onset of illness or neglect. Critically ill prisoners may experience delayed referrals to external medical services, leading to deaths en route or shortly after hospital admission, while the underlying causes, neglected disease, inadequate care, or injury, originate in custody [20,21]. Moreover, administrative incentives may encourage reporting deaths as occurring outside prison (in transit or hospital) to avoid scrutiny or legal consequences associated with in-custody mortality [20].

Among the deceased, 12 individuals (35.29%) were convicted prisoners, four (11.76%) were undertrial prisoners, and two (5.88%) died in police custody. For 16 individuals (47.05%), the legal status was not mentioned in the inquest papers, reflecting serious gaps in documentation and procedural transparency. Inquest reports provided by police often lacked essential details, such as whether the deceased was under trial, convicted, or in police custody. Missing information included the legal sections under which the individual was imprisoned, the duration of custody, and the circumstances of confinement. The absence of these details poses significant challenges for autopsy surgeons. Without information on incarceration period or charges, it is difficult to correlate injuries, disease progression, or possible ill-treatment with the custodial context [21]. Although police are not legally bound to include legal status in inquest reports, such information would be highly useful for forensic evaluation. These omissions hinder accurate medical interpretation and raise concerns about transparency, accountability, and custodial record-keeping integrity.

Most deaths, 31 of 34 cases (91.17%), were classified as natural. Three deaths (8.82%) were categorized as unnatural, attributed to blunt trauma during confrontation or to injuries present before arrest. Numerous studies suggest underreporting and misclassification of unnatural causes. Factors such as administrative pressure, lack of independent autopsies, and conflicts of interest may result in injuries indicative of violence or neglect being downplayed or reclassified as natural or accidental [22,23]. In practice, when blunt force trauma is identified, inquest reports frequently attribute the injuries to “confrontation” with police or suggest they predated custody. This narrative minimizes institutional culpability and is not always corroborated by independent forensic analysis [23].

Regarding comorbidities, 13 individuals (38.23%) had hypertension and 11 (32.35%) had diabetes, consistent with other studies [15,17]. The leading specific medical causes of death were CAD: nine cases (26.4%); chronic liver disease: four cases (11.76%), septicemia: eight cases; COVID-19: two cases (5.88%); and pneumonia: one case (2.94%). Other isolated causes included brain injury, hypertrophic cardiomyopathy, lung carcinoma, tuberculosis, spinal injury, intestinal and esophageal perforation, chronic lung disease, and meningoencephalitis. The high rate of CAD-related deaths reflects not only background risks but also structural and healthcare deficiencies. Prisoners in India often belong to socioeconomically marginalized groups with higher burdens of cardiovascular risk factors such as tobacco use, hypertension, diabetes, poor nutrition, and psychosocial stress [20,24,25]. Multiple studies have identified CAD as the leading cause of death among inmates, reflecting both the vulnerabilities of this population and failures in preventive care within custodial settings. Addressing this issue requires strengthening prison health systems and broader public health interventions, in line with constitutional guarantees of the right to life and health [20,22,24,26]. By contrast, studies from other states in India have reported tuberculosis as a common natural cause of custodial death [27-30].

Several measures can help prevent custodial deaths. Jail inmates must only be held in official detention centers; secret detention should be strictly prohibited. Basic records (arrest details, health status, responsible officers, and interrogations) must be properly documented and made available for review. Prisoners should have prompt and regular access to doctors, lawyers, and, when possible, family members. Authorities must carry out unannounced visits when torture or ill-treatment is suspected. Regular internal and external inspections should be conducted, including by health and safety bodies, the ombudsman, human rights agencies, and non-governmental organizations. Confessions obtained through torture or ill-treatment must not be admissible in court. Effective measures should be implemented to prevent prisoner-on-prisoner violence (e.g., separating groups by age, sex, or criminal record). Laws should allow detainees to file complaints if threatened or abused, and such complaints must be investigated impartially. All staff handling detainees must be trained to prevent arbitrary killings, torture, and ill-treatment, and rules must explicitly prohibit such acts [31].

## Conclusions

Critical gaps exist in the justice and prison systems, particularly in ensuring the right to life and healthcare for individuals in custody. While many deaths are officially classified as natural, systemic issues such as delayed medical intervention, inadequate healthcare infrastructure, and poor documentation suggest that some may be preventable. Urgent reforms are needed, including improved prison healthcare services, timely referrals, independent investigations, and stricter adherence to legal and human rights standards. Ensuring the dignity and safety of individuals in custody is both a constitutional obligation and a measure of a just and humane society.
